# Structural and biochemical insights of CypA and AIF interaction

**DOI:** 10.1038/s41598-017-01337-8

**Published:** 2017-04-25

**Authors:** Biancamaria Farina, Gianluigi Di Sorbo, Angela Chambery, Andrea Caporale, Guido Leoni, Rosita Russo, Fabiola Mascanzoni, Domenico Raimondo, Roberto Fattorusso, Menotti Ruvo, Nunzianna Doti

**Affiliations:** 1 Istituto di Biostrutture e Bioimmagini, C.N.R. and CIRPEB, Via Mezzocannone 16, 80134 Napoli, Italy; 2 Dipartimento di Scienze e Tecnologie Ambientali, Biologiche e Farmaceutiche, Università degli Studi della Campania Luigi Vanvitelli, via Vivaldi 46, 81100 Caserta, Italy; 3 Nouscom s.r.l. via di Castel Romano 100, 00128 Roma, Italy; 4 Sapienza, Università di Roma- Viale Regina Elena 324, 00161 Roma, Italy

## Abstract

The Cyclophilin A (CypA)/Apoptosis Inducing Factor (AIF) complex is implicated in the DNA degradation in response to various cellular stress conditions, such as oxidative stress, cerebral hypoxia-ischemia and traumatic brain injury. The pro-apoptotic form of AIF (AIF(Δ1-121)) mainly interacts with CypA through the amino acid region 370–394. The AIF(370-394) synthetic peptide inhibits complex formation *in vitro* by binding to CypA and exerts neuroprotection in a model of glutamate-mediated oxidative stress. Here, the binding site of AIF(Δ1-121) and AIF(370-394) on CypA has been mapped by NMR spectroscopy and biochemical studies, and a molecular model of the complex has been proposed. We show that AIF(370-394) interacts with CypA on the same surface recognized by AIF(Δ1-121) protein and that the region is very close to the CypA catalytic pocket. Such region partially overlaps with the binding site of cyclosporin A (CsA), the strongest catalytic inhibitor of CypA. Our data point toward distinct CypA structural determinants governing the inhibitor selectivity and the differential biological effects of AIF and CsA, and provide new structural insights for designing CypA/AIF selective inhibitors with therapeutic relevance in neurodegenerative diseases.

## Introduction

Key players of programmed cell death (PCD) signalling pathways also contribute to acute neurological insults such as hypoxic-ischemic brain damage (HI), traumatic brain injury (TBI) and stroke^1, 2^. A critical step of the apoptotic cascade is the permeabilization of the outer mitochondrial membrane (OMM), mediated by pro- and anti-apoptotic Bcl-2 family proteins, culminating in the release of soluble intermembrane proteins from mitochondria^1–3^. Once released into the cytosol, these mitochondrial proteins activate caspase-dependent and/or caspase-independent cell death pathways^4^. Caspase-dependent cell death, mediated by the release of cytochrome C from mitochondria, apoptosome formation and caspase activation, has been studied in detail^5^. Instead, very little is known about the contribution of caspase-independent mechanisms to neuronal cell death in neurodegenerative diseases^6^.

Apoptosis-Inducing Factor (AIF) is one of the pro-apoptotic mitochondria-released factors, which contributes to apoptotic nuclear DNA damage in a caspase-independent way^6^.

AIF is an old mitochondrial flavoprotein implicated in embryonic development and cardiac cell survival^7^. In healthy mitochondria, the mature form of AIF (AIF(Δ1-101)), containing two FAD-binding domains (residues 122–262 and 400–477), a NADH-binding domain (residues 263–399) and a C-terminal domain (residues 478–613), is anchored to the inner mitochondrial membrane (IMM), where it plays a bioenergetic role by regulating mainly the activity of the mitochondrial respiratory chain complex I^7, 8^. Furthermore, AIF induces cell-death in response to various cellular stress conditions such as oxidative stress^9^, DNA damage^10^, cerebral hypoxia-ischemia^11^ and TBI lethal mechanisms^12^. The multiple intracellular stress pathways at the end converge on mitochondria depolarization and fragmentation^1^ and the consequent release of the apoptogenic form of AIF (AIF(Δ1-121)) from mitochondria to nuclei, where it triggers chromatin condensation and large scale DNA fragmentation through a mechanism independent of caspases activation^9–12^. The pro-apoptotic function of AIF is tightly regulated in neurons, and involves its association with the Cyclophilin A (CypA) protein^13, 14^.

CypA is an abundant, ubiquitously expressed protein, first discovered as an intracellular receptor of the immunosuppressive drug cyclosporin A (CsA)^15^. CypA exerts peptidyl-prolyl-isomerase activity *in vitro*, suggesting that it can affect conformational changes of other proteins in cells; however its function is largely unknown^15^. Moreover, several evidence suggested its involvement in key processes underling human pathologies^16^. Beyond the different roles played into different cellular contexts, CypA promotes the lethal action of AIF by mediating its nuclear translocation and/or its DNase activity, depending on the cell type and/or the apoptotic insult^13, 14, 17^. Loss or down-regulation of AIF and CypA, combined with caspases inhibition, has been associated with additive neuroprotection effects in several *in vivo* animal models, through a not yet clarified mechanism^12–14^.

Despite many evidences indicate the complex as a key player of neuronal loss pathways, very little is known about the molecular interactions between the two targets, since the three-dimensional (3D) complex structure is not available so far. To date, only a molecular docking model, which fits with mutational studies, has been proposed by Candé and coworkers^13^. In this model, AIF contributes to the interface with α11 (P345-R358), with β-strands (T328-F334, V361-N366, R387-A397) and several turns (E359-G360, A367-S371, K382-G386) that form together a β-sheet bulge. CypA contributes to the interface with α3 (M136-G146), the following turn and the β-strand β8 (S147-G150), as well as part of its central β-barrell comprising β3-β4 (R55-Q63), β4-β5 (H92-A101) and β6-α2 (Q111-T119). On the basis of this model, we have identified the peptide AIF(370-394) as the minimal region of AIF that interacts with CypA. However, other regions supposed to be involved in the mutual interaction, have failed to completely validate the model^13^.

The peptide AIF(370-394) identified is able to inhibit the AIF(Δ1-121)/CypA complex formation *in vitro* by binding CypA with a relatively affinity in the low micromolar range. Moreover, the delivery of the peptide in neuronal cells provided a pronounced neuroprotection in a model of AIF(Δ1-121)/CypA-mediated glutamate-induced cell death^18^, by blocking the nuclear translocation of the two proteins^18^. These findings further demonstrated that the direct inhibition of the AIF(Δ1-121)/CypA complex formation has a therapeutic relevance for neurodegenerative diseases treatment and showed that AIF(370-394) may represent a valid ligand model to study the interaction between AIF and CypA and to design new and more effective inhibitors.

On this framework, in the present work, the AIF(Δ1-121) and AIF(370-394) interacting regions on CypA have been mapped by a combination of Nuclear Magnetic Resonance (NMR) spectroscopy and mass spectrometry (MS) and the minimal CypA-region mainly involved in the interaction with AIF has been identified through binding assays of representative CypA peptides. Isothermal titration calorimetry (ITC), has been also used to assess the interactions observed by NMR and to compare the binding mechanism of AIF(370-394) with that of the known inhibitor CsA. Finally, data have been used to build a docking model of the AIF/CypA interaction. Overall, our study identifies a hydrophilic CypA surface recognized by AIF(Δ1-121), as well as by AIF(370-394), that represents a new and selective druggable region of CypA.

## Results

### Mapping of AIF(Δ1-121) binding site on CypA by NMR-CSP studies

**Figure 1:**
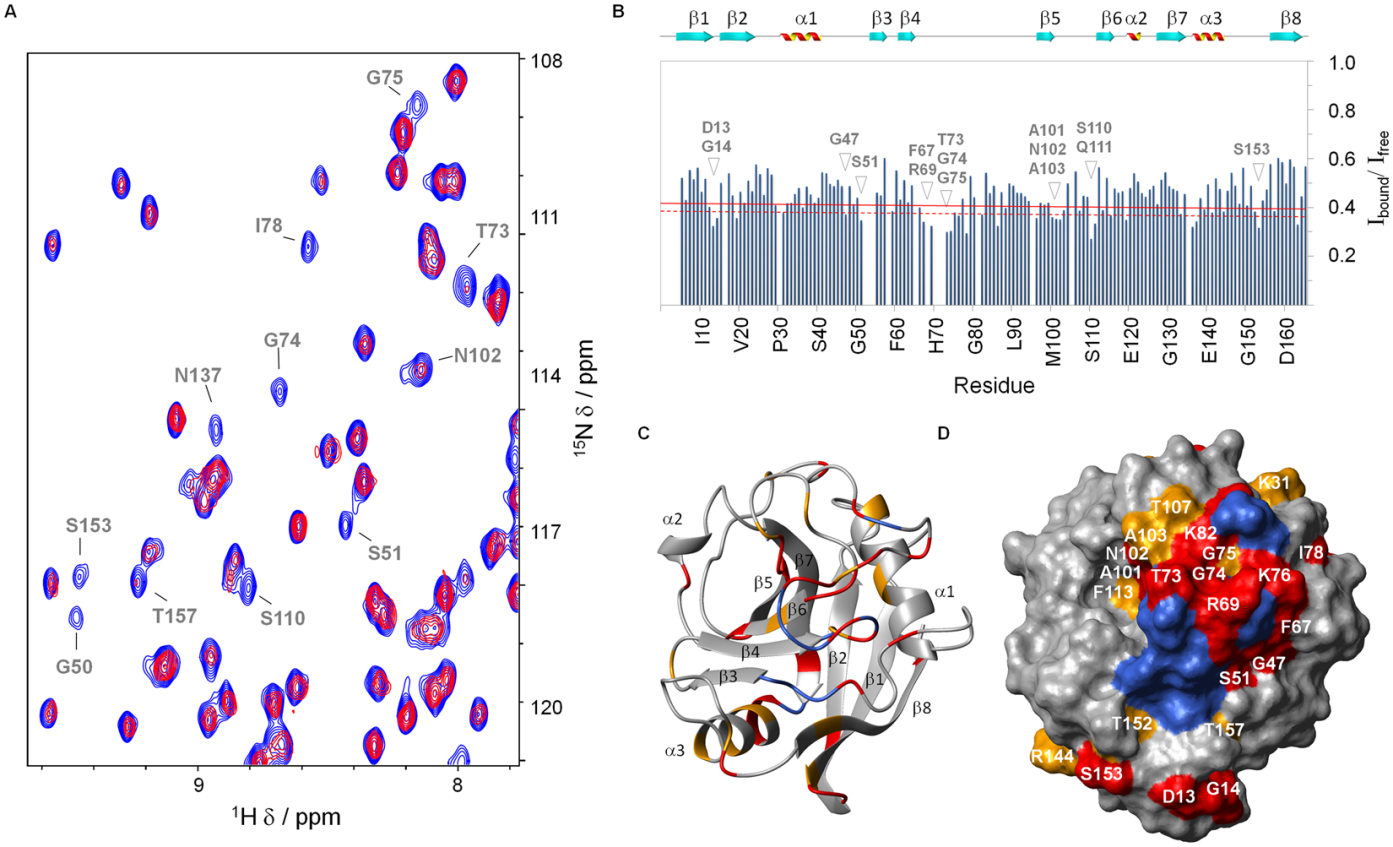
NMR CSP analysis of CypA/AIF(Δ1-121) interaction. (**A**) Superposition of a 2D [^1^H, ^15^N] HSQC section of ^15^N-CypA in the absence (blue) and in the presence of 0.6 equivalents of AIF(Δ1-121) (red). Amide cross-peaks with significant intensity reduction are indicated. (**B**) Bar graphs of the ratio of the intensity of CypA in presence of 0.6 eq. AIF(Δ1-121) (I_bound_) and free CypA (I_free_) as a function of the amino acid residue. The mean value minus 0.5 times the standard deviation (SD) is shown as a continuous line; the mean value minus 1 SD as a broken line. The secondary structure elements are also indicated. (**C**,** D**) CSP mapping onto the representative conformer of the NMR structure of CypA (PDB ID 1OCA)^28^ shown as ribbon drawing and as solvent-accessible surface, respectively. Residues for which I_bound_/I_free_ is less than the mean minus 1 SD or is included between the mean minus 1 SD and the mean minus 0.5 SD are shown in red and in orange, respectively. Residues having cross-peaks totally or scarcely visible in the HSQC: C52 (loop α1-β3), F53, H54 (sheet β3), G65, T68, H70, N71, G72, E81 (loop β4-β5), G135 (loop β7-α3), were excluded from the CSP analysis and are painted in navy on the CypA structure. Pictures **C** and **D** were prepared using the software MOLMOL^42^.

To identify CypA residues involved in the interaction with AIF(Δ1-121), we performed a NMR Chemical shift perturbation (CSP) analysis by acquiring 2D [^15^N, ^1^H] HSQC spectra of ^15^N-labeled CypA in the absence and presence of increasing concentrations of unlabeled AIF(Δ1-121). Upon addition of AIF(Δ1-121), a dose-response reduction of cross-peaks intensities was observed in the 2D [^15^N, ^1^H] HSQC spectrum of CypA. The cross-peaks virtually disappeared upon addition of a stoichiometric amount of AIF(Δ1-121) (ratio 1:1). This behavior is in agreement with the formation of a large complex, in a slow-intermediate exchange regime between free and bound protein, as expected from the K_D_ value in the low micromolar range (0.9 μM)^18^. At sub-stoichiometric levels of AIF(Δ1-121) (until a ratio 1:0.6, CypA:AIF(Δ1-121)), differential HSQC cross-peaks intensity reduction provided indications on the residues of CypA involved in the interaction with AIF(Δ1-121) (Fig. 1A). Intensity changes of amide signals (I_bound_/I_free_) following protein addition are plotted in the histogram shown in Fig. 1B. The mean value of I_bound_/I_free_ was ~0.4, indicating that the fraction of bound protein is ~0.6, in agreement with the 1:0.6 CypA:AIF(Δ1-121) ratio and the 1:1 stoichiometry of the complex^18^. Residues having the largest intensity reduction (higher than the mean value plus 1 SD) are located in the loop β1-β2 (D13, G14), sheet β2 (R19), loop α1-β3 (G47, S51), loop β4-β5 (F67, R69, T73, G74, K76, I78, K82, E86, G96), sheet β5 (M100), loop β5-β6 (A101, N102, S110, Q111), sheet β6 (C115), loop β6-α2 (T119), sheet β7 (K133), helix α3 (M136, N137), loop α3-β8 (S153), sheet β8 (Q163). Residues having ΔI included between 0.5 and 1 SD over the mean value, are located in the sheet β1 (V12), helix α1 (K31, F36), loops β3-β4 (G59), β4-β5 (D66, G75, F88) and β5-β6 (A103, T107), sheet β6 (F113), helix α3 (V139, A141, R144), loop α3-β8 (T152), sheet β8 (T157). Mapping of these residues onto the structure of CypA protein (Fig. 1C and D) reveals that AIF(Δ1-121) binding affects a rather contiguous and extensive surface, constituted essentially by the long β4-β5 loop, the β5 and β6 sheets and their connecting loops (Fig. 1C and D).

### Mapping of the AIF(370-394) ligand-binding site on CypA by NMR-CSP studies

To further understand the basis of the specific interaction between CypA and AIF(Δ1-121), we performed a CSP analysis following the H^N^ chemical shift variations of the ^15^N-CypA signals induced by addition of AIF(370-394).

**Figure 2:**
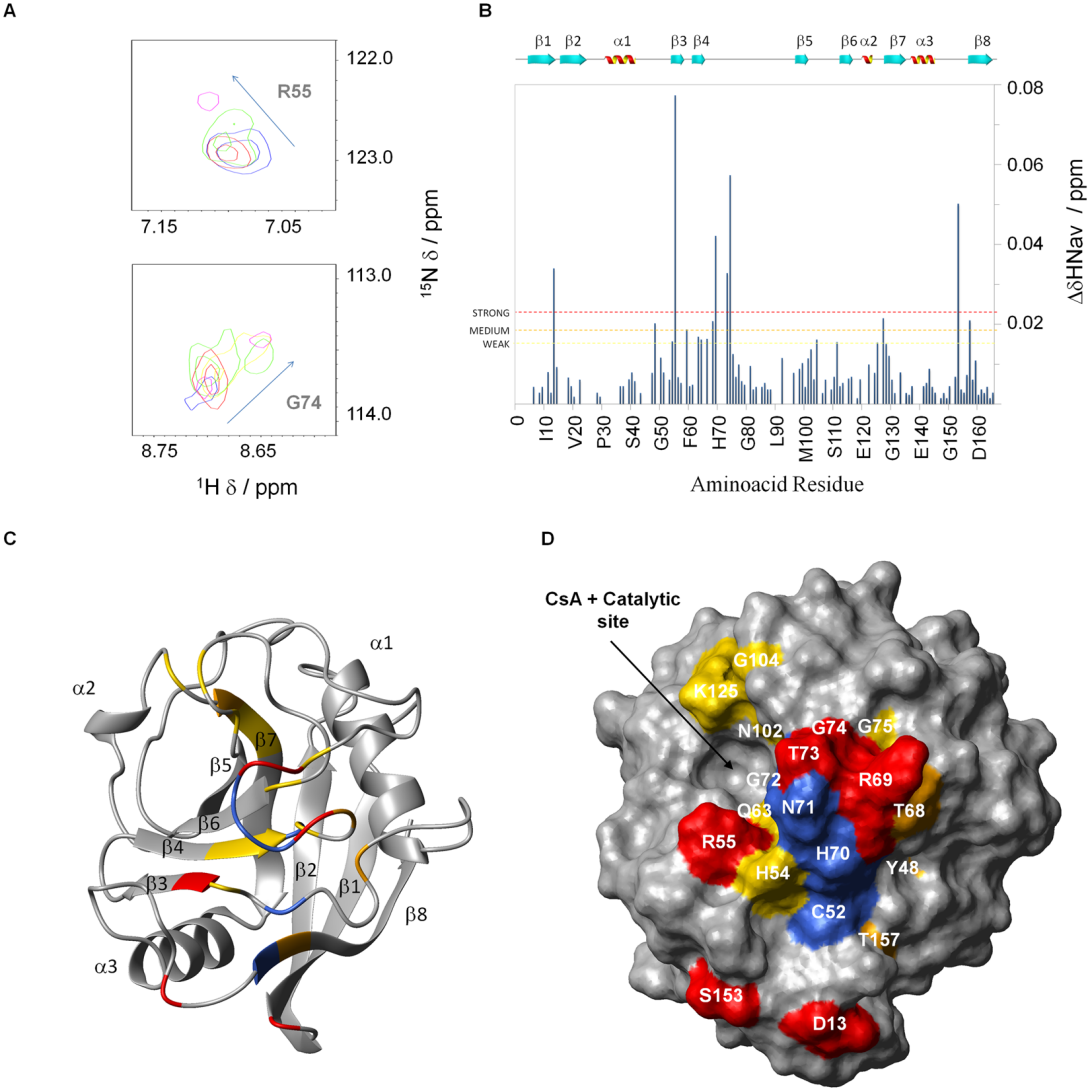
Identification of the AIF(370-394) interaction surface on CypA by NMR-CSP studies. (**A**) Superposition of two 2D [^1^H, ^15^N] HSQC section of ^15^N-CypA in the absence (blue) and in the presence of different equivalents of AIF(370-394), 1 (red), 2 (green), 5 (yellow) and 10 (magenta), respectively. (**B**) Bar graphs of the average combined chemical shift difference (ΔHN_av_) as a function of the amino acid residue. Mean plus 2 SD, 1 SD and 0.5 SD ΔHN_av_ values are indicated by red, orange and yellow broken lines, respectively. The secondary structure elements are also shown. (**C**,** D**) CSP data were mapped onto a ribbon drawing and solvent accessible surface of the representative conformer of the CypA NMR structure (PDB ID 1OCA)^28^ and showed with a color gradient from yellow (weak) to orange (medium) to red (strong). Residues having cross-peaks totally or scarcely visible in the HSQC (C52 of the loop α1-β3; G65, H70, N71, G72 of the loop β4- β5; G135 of the loop β7-α3) were excluded from the CSP analysis (**B**) and are painted in navy on the CypA structure. Pictures **C** and **D** were prepared using the software MOLMOL^42^.

A number of resonances in the 2D ^1^H -^15^N-HSQC spectrum exhibited continuous and significant chemical shift variations following the titration of ^15^N-CypA with AIF(370-394) up to a 10-fold molar excess. Most changes occurred in the intermediate exchange regime on the NMR time-scale (Fig. 2A), according with the KD in the low micromolar range^18^. CSPs were quantified in terms of ΔδHN_av_s and classified as strong, medium and weak (see Methods section for details).

Strong CSP effects on CypA occurred on the sheet β3 (R55) and on the loops β1- β2 (D13), β4-β5 (R69, T73, G74), α4-β8 (S153), medium CSPs on the loops α1-β3 (Y48), β4-β5 (T68), α2- β7 (V127) and on the strand β8 (T157), and weak CSPs on the sheets β3 (H54) and β4 (Q63, G64), the loops β4-β5 (D66, G75), β5-β6 (N102, G104, Q111) and α2-β7 (K125, V128, F129) (Fig. 2B). These residues are mapped onto the ribbon structure and the surface of CypA (Fig. 2C and D).

Results thus show that residues involved in the interaction are mainly located on a side of CypA protein constituted by the loops connecting β-strands, close to the catalytic site of CypA (Fig. 2D). This region is largely characterized by polar and charged residues (Supplementary Fig. S1A), suggesting that hydrophilic interactions mostly drive the binding of AIF(370-394) to CypA.

Interestingly, the CypA surface in contact with AIF(370-394) almost entirely overlaps with that covered by the full-length protein AIF(Δ1-121), suggesting that the peptide and the protein share the same binding region on CypA, according to competition experiments reported in literature^18^.

### NMR-hydrogen/deuterium exchange experiments

**Figure 3:**
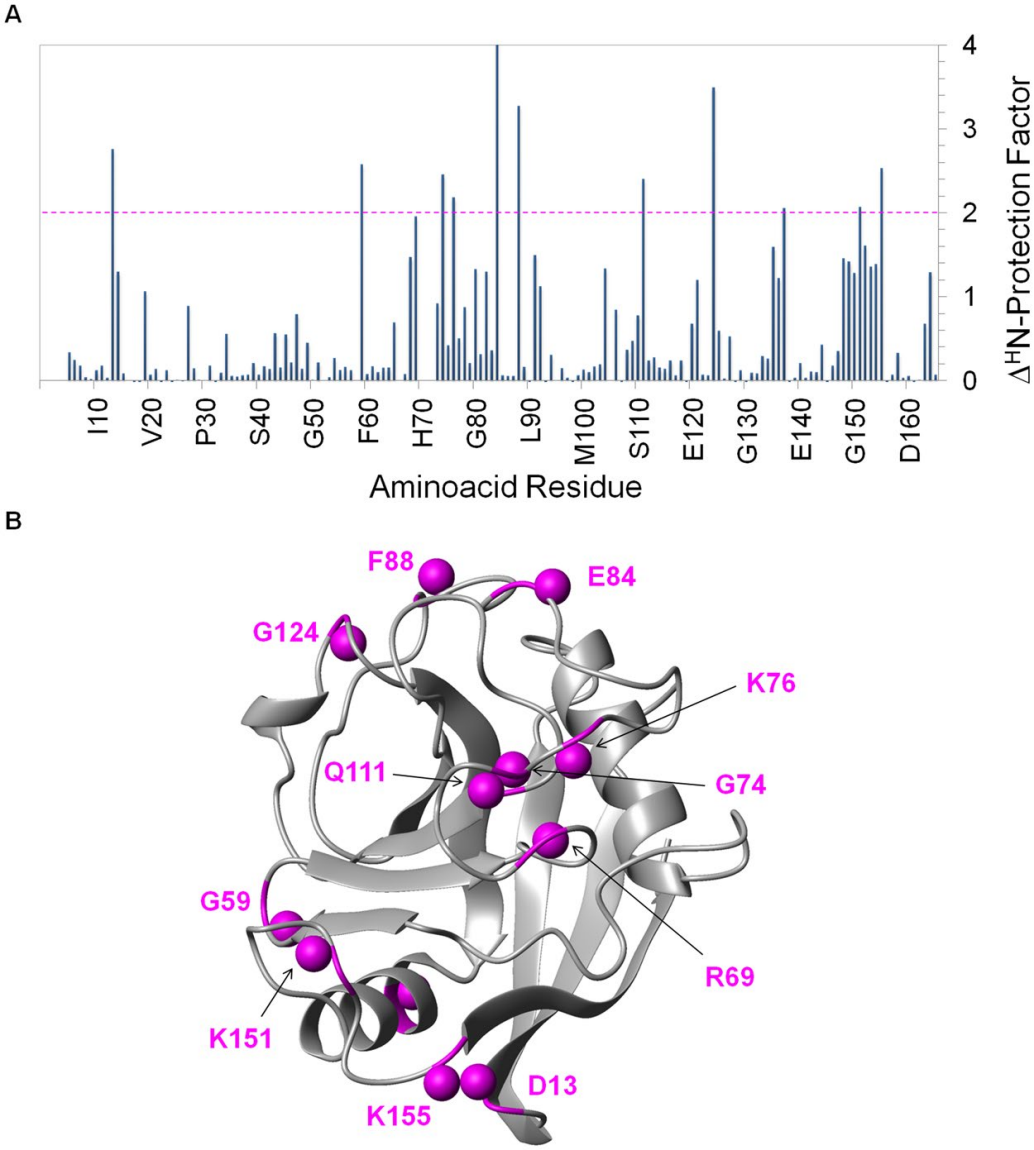
Identification of the AIF(370-394) interaction surface on CypA by NMR-H/D exchange studies. (**A**) Bar graphs of the difference of the H^N^-PFs between the free and bound protein (ΔH^N^-PF) as a function of the amino acid residue. The broken line traced at ΔH^N^-PF = 2 is used as threshold to identify the protected amides upon binding. (**B**) Amide hydrogens of residues showing ΔH^N^-PFs higher than 2 were represented as balls in magenta onto the ribbon drawing of the representative conformer of the NMR structure of CypA (PDB ID 1OCA)^28^. (**C**) Comparison of the ΔH^N^-PF and CSP patterns by mapping on the amino acid sequence of CypA. Residues affected upon AIF(370-394) binding both by the H/D exchange and by the CSP studies are shown in blue; residues identified by CSP or H/D exchange studies are shown in red and in magenta, respectively.

A complementary mapping of the AIF(370-394) binding site on CypA was carried out by performing hydrogen/deuterium (H/D) exchange experiments of ^15^N-labeled protein. The protein was thus incubated with and without the ligand in both D_2_O and H_2_O, followed by the acquisition of 2D ^1^H-^15^N HSQC spectra. The intensity of several HSQC cross-peaks of CypA significantly increased in the presence of AIF(370-394) compared to those in the isolated protein, as expected when amide protons are sequestered into binding sites (Supplementary Fig. S2A and B). Changes were quantified in terms of the amide proton protection factors (H^N^-PF) and plotted versus each residue (Supplementary Fig. S2C and D)^19^. To better evaluate the residues with an increased protection factor, the difference of the H^N^PFs between the free and bound protein in D_2_O (ΔH^N^-PF) was calculated, plotted versus the residue number and mapped onto the three-dimensional structure of CypA (Fig. 3A and B). A strong effect (ΔH^N^-PF > 2) was determined for residues located in the loops β1-β2 (D13), β3-β4 (G59), β4-β5 (R69, G74, K76, E84, F88), β5-β6 (Q111) and α2-β7 (G124), in the helix α3 (N137), and in the loop α3-β8 (K151, K155) (Fig. 3B). As shown in Fig. S3, the ΔH^N^-PF pattern overlaps well with the interaction region identified by CSP studies, confirming the localization of the AIF(370-394) binding site on the β-sheet surface of CypA. However, there are some residues, including K76, E84, F88, G124, N137, K151 and K155, with increased H^N^-PFs in presence of AIF(370-394) not found in CSP studies. All these residues (except N137) belong to loop regions contiguous or proximal to the binding site, suggesting that the H^N^-PFs increase could be due to a reduced conformational flexibility of unstructured regions upon ligand interaction. Among these, residues K151 and K155 create a more extended and continuous AIF(370-394) interaction surface (Supplementary Fig. S3).

### Enzymatic MS-based foot-printing

**Figure 4:**
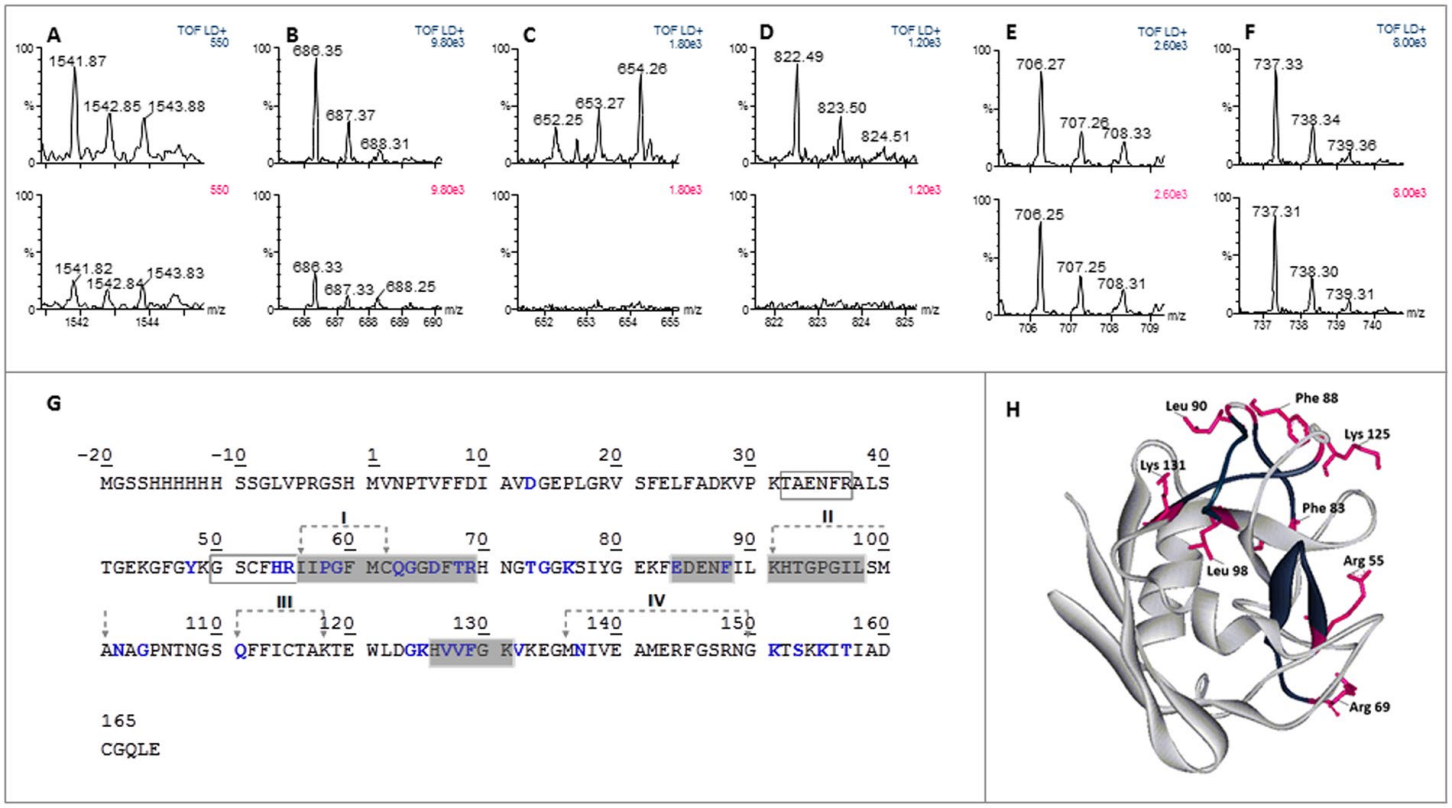
MALDI-TOF spectra of tryptic (**A**, **B**) and chymotryptic (**C**,** D**) CypA peptides protected from proteolysis following hydrolyses performed in the absence (upper panel) and in the presence (lower panel) of AIF(370-394) peptide. Representative CypA control peptides for which no relative intensity changes were detected are reported in (**E)** (sequence region 50-55) and (**F)** (sequence region 32-37). (**G**) Tryptic and chymotryptic peptides protected from proteolysis in the presence of AIF(370-394) peptide are grey shaded on CypA sequence. Amino acids identified by CSP-NMR and/or H/D-exchange are reported in blue. Moreover, the regions I-IV identified by Candé *et al*.^13^ are indicated on the sequence. Control tryptic peptides showed in **E**, **F** are boxed. (**H**) Tryptic and chymotryptic peptides protected from proteolysis in the presence of AIF(370-394) peptide and amino acids in the close proximity of these peptides are also highlighted in the CypA structure (PDB ID 1OCA)^28^ in blue and pink, respectively.

In order to support NMR results and provide additional experimental evidences of the interaction surfaces of the CypA/AIF(370-394) complex, we probed the accessible surfaces of CypA in the presence and absence of AIF(370-394) by MS analysis. By this approach, ligand-induced protection against proteolysis is used to map sites stabilized in protein complexes (protein foot-printing)^20^. Samples of free CypA protein and CypA/AIF(370-394) mixtures were subjected to trypsin and chymotrypsin hydrolysis. As shown in Fig. 4A, the relative intensity of tryptic peptides I56-R69 (m/z 1541.87, Fig. 4A, upper panel) and H126-K131 (m/z 686.35, Fig. 4B, upper panel) was found to be strongly reduced when hydrolyses were performed in the presence of AIF(370-394) (Fig. 4A and B, lower panels, respectively). These results indicate that basic residues R55 and/or R69 and K125 and/or K131 were protected from proteolysis upon AIF(370-394) binding (Fig. 4G and H). However, similarly to what observed for an internal control sequence T32-R37 (Fig. 4F), no relative intensity changes were detected for the peptide G50-R55 in the presence of the ligand (Fig. 4E), strongly suggesting that R69 is the basic amino acid of the I56-R69 peptide protected upon AIF(370-394) binding. Both regions mapped by trypsin MS-based foot-printing fall within the CypA interaction surface identified by NMR studies. Moreover, our data are in partial agreement with the 3D molecular model of the CypA/AIF(Δ1-121) complex proposed by Candé and coworkers^13^. Indeed, the tryptic peptide I56-R69 is located within the AIF interacting region I predicted by the model (Fig. 4G)^13^. On the contrary, the region H126-K131 is not included in those proposed by the 3D model. Furthermore, for these residues, included in the loop α2-β7, NMR-CSP studies indicate that they are weakly perturbed in presence of AIF(370-394), suggesting that they could be not directly involved in the interaction but could instead undergo a conformational rearrangement following the interaction with AIF(370-394), as recently suggested by a computational and NMR study^21^.

Additional regions protected from proteolysis were identified by chymotrypsin MS foot-printing. Indeed, intensities of consecutive peptides E84-F88 (m/z 652.25, Fig. 4C, upper panel) and K91-L98 (m/z 822.49, Fig. 4D, lower panel) were found also strongly decreased in the presence of the peptide ligand. Accordingly, residues included in the region E84-F88 resulted strongly protected in the NMR H/D exchange studies, although they were not identified by NMR-CSP experiments, as shown above. Also, no residues of segment K91-L98 were identified by NMR-CSP and H/D exchange studies. This stretch is located upstream a region containing several amino acid identified by NMR analysis, suggesting a possible contribution to the interaction through conformational rearrangements, a hypothesis largely corroborated also by the studies of Doshi *et al*., indicating a relevant structural flexibility in this region upon perturbation by substrate binding or distant mutations^21^. In addition, this region is included in region II (H92-A101) suggested by the predicted molecular model of Candè *et al*.^13^. Further experiments are described below to clarify this specific point.

### Design and testing of CypA-derived synthetic peptides

NMR and MS data have shown that the AIF binding region on CypA is extensive and discontinuous. To gain further insights on the CypA regions mostly involved in the interaction with AIF, a set of overlapping CypA-peptides was generated and tested for binding against the protein and the AIF(370-394) fragment. Although peptide-peptide interaction insights might be prejudiced by the lack of appropriate 3D structures and require additional experimental assessment with full length proteins, they can mimic and anticipate in several cases real interactions^22^. The binding affinity toward AIF(370-394) was thus assessed through direct binding assays using the EnSpire-label free system (see Supplementary Information for details)^23^. K_D_ values determined by this approach are reported in Table 1 and the binding curves are shown in Supplementary Fig. S4. Remarkably, most of CypA-derived peptides provided a K_D_ in the low micromolar range, which is comparable to that of the full length CypA/AIF(370-394) complex^18^. Among these peptides, CypA(55-69) exhibited the strongest affinity for AIF(370-394) (K_D_ = 6.3 ± 0.1 μM). Furthermore, a significant positive effect in terms of KD was observed by combining peptides CypA(55-69) with the lower affinity N-terminal overlapping peptide, CypA(49-62). In addition, when CypA(67-79) was tested in combination with the peptide CypA(49-62), distant in the primary sequence but in close proximity in the tertiary structure, a significant increase of affinity for AIF(370-394) was detected. This finding further confirmed the importance of the CypA region encompassing residues K49-Y79, belonging to loops α1-β3, β3-β4 and β4-β5, in the interaction with the AIF peptide.

**Table 1:** Comparison of AIF(Δ1-121) and AIF(370-394) binding affinities for the CypA peptides studied.

Peptides	AIF(370-394) K_D_ (μM)	AIF(Δ1-121) K_D_ (μM)
CypA(3-27)	>10^3^	4.2 ± 1.2
CypA(49-62)	123 ± 5.8	25.9 ± 4.2
CypA(55-69)	6.3 ± 1.0	10.3 ± 1.6
CypA(67-79)	>10^3^	47.3 ± 6.7
CypA(83-90)	No binding	No binding
CypA(99-109)	>10^3^	26.5 ± 2.8
CypA(121-131)	18.4 ± 1.8	19.3 ± 1.6
CypA(136-150)	No binding	No binding
CypA(154-165)	No binding	No binding
CypA(55-69) + (67–79)	34.9 ± 8.1	51.4 ± 12.2
CypA(55-69) + (49–62)	4.6 ± 1.3	2.2 ± 0.7
CypA(67-79) + (49–62)	3.1 ± 1.3	2.8 ± 0.9
CypA(55-69) + (67–79) + (49-62)	20.7 ± 6.5	25.2 ± 5.0
CypA(121-131) + (99–109)	43 ± 6.8	27.2 ± 5.8
CypA(121-131) + (83–90)	87 ± 5.8	31.3 ± 2.8
CypA(99-109) + (83–90)	304 ± 10.8	No binding
CypA(121-131) + (99–109) + (83-90)	268 ± 9.65	38.8 ± 4.6
CypA(55-69)R55	No binding	42.1 ± 9.8
CypA(55-69)T68	41.5 ± 9.65	43.2 ± 8.8
CypA(55-69)R69	No binding	>10^3^
CypA wild type	3.0 ± 0.60	1.6 ± 0.1
CypA^R55A^	3.1 ± 0.70	1.6 ± 0.2

Peptides deriving from region 83–150 showed an affinity for AIF(370-394) lower than that of CypA(55-69), suggesting a minor involvement of this region in the interaction (Table 1 and Figure S4 in the Supporting Information). Among the peptides from this region, CypA(121-131) appeared as the best binder with a K_D_ of about 24 μM. Moreover, the combination of CypA(121-131) with CypA(83-90) and CypA(99-109), covering the C-terminal portion of loop β4-β5, and strand β5 did not provide an increase of affinity for CypA. This observation, together with data on protein dynamics, suggested that region 83–150 may contain a second lower affinity binding site across residues 121–131 and that residues 95–110, upon AIF binding, may undergo a significant allosteric rearrangement due to the intrinsic conformational flexibility of the protein in this region^21^.

Peptides CypA(3-27), CypA(136-150), designed on the basis of the previous model of the AIF(Δ1-121)/CypA complex^13^, and CypA(154-165), did not interact with AIF(370-394), in agreement with MS data (Supplementary Fig. S4).

CypA-derived peptides were also tested against AIF(Δ1-121) protein (Table 1 and Supplementary Fig. S5). Noteworthy, a very good correlation was observed between the K_Ds_ measured for AIF(370-394) and AIF(Δ1-121). An overall significant increase in the CypA-AIF(Δ1-121) affinity was observed for peptides CypA(3-27), CypA(99-109) and CypA(49-62) (Table 1), suggesting that additional interactions, outside the 370–394 region, stabilize the complex with AIF(Δ1-121).

To dissect the relative contributions of crucial residues of CypA to the binding with AIF, a series of mutated CypA-peptides were synthesized. K_D_ values are reported in Table 1 and binding curves in Supplementary Figs S4 and S5.

In particular, residues R55, T68 and R69, supposed to play a key role in the interaction between CypA(55-69) and AIF(370-394) by NMR data, were replaced by alanine. Data show that the affinity of the alanine variants for AIF(370-394) and AIF(Δ1-121) was largely reduced, confirming the importance of these residues in the recognition. In particular, replacement of R69 of CypA with alanine completely abrogated the binding to both the AIF forms, the R55 replacement completely abrogated the affinity with AIF(370-394), and slightly reduced that with AIF(Δ1-121), whereas T68 replacement decreased the affinity by 7- and 10-fold for AIF(370-394) and AIF(Δ1-121), respectively.

Altogether, data demonstrate that the interaction site of AIF(370-394) on CypA is close to its catalytic site and partially interacts with the CsA-binding residues and in particular with the catalytic residue R55^24^.

However, several evidences demonstrate that CsA binding on CypA does not prevent the interaction with AIF(Δ1-121)^13^ (see also Supplementary Fig. S6) and that the lethal activity of the AIF(Δ1-121)/CypA complex is completely independent from the PPIase activity of CypA^13^. Also, the binding of AIF(370-394) to CypA does not alter the catalytic activity of CypA^18^. To strengthen this concept we measured the affinity of the R55A CypA mutant (CypA^R55A^) for AIF peptide and protein by EnSpire-label free techniques^23^. As shown in Fig. S7A and **B**, AIF(370-394) and AIF(Δ1-121) bound CypA and CypA^R55A^ in a similar way suggesting that, although R55 does interact with the peptide to some extent, its role in the interaction is very limited.

On the other hand, the inability of the AIF(370-394) peptide to inhibit the cis-trans prolyl peptidyl isomerase activity of CypA can be justified by the generally higher affinity of CypA for its catalytic substrates. We have tested this hypothesis using a CypA peptide substrate^25^ and performing binding experiments between wild type and R55A mutant protein and the substrate in the presence and absence of the AIF(370-394) and CsA. Data showed that, in line with the large difference in affinity (57 nM for the substrate and 6 μM for the AIF peptide), AIF(370-394), used at a 10-fold excess, is unable to prevent the binding of both CypA and CypA^R55A^ to the substrate, but recognition is abolished in the presence of the strong inhibitor CsA (Supplementary Fig. S8). Remarkably, binding of the substrate in the presence of larger excess of AIF(370-394) does impair the enzyme-substrate recognition (not shown).

Furthermore, a substitution of the catalytic residue H126^24^ in CypA(121-131) with alanine, as expected from the CSP-data, did not affect significantly CypA binding for both the peptide and the full-length protein, further remarking the concept that the CypA catalytic pocket is not involved in the interaction with AIF.

### Thermodynamic studies

**Figure 5:**
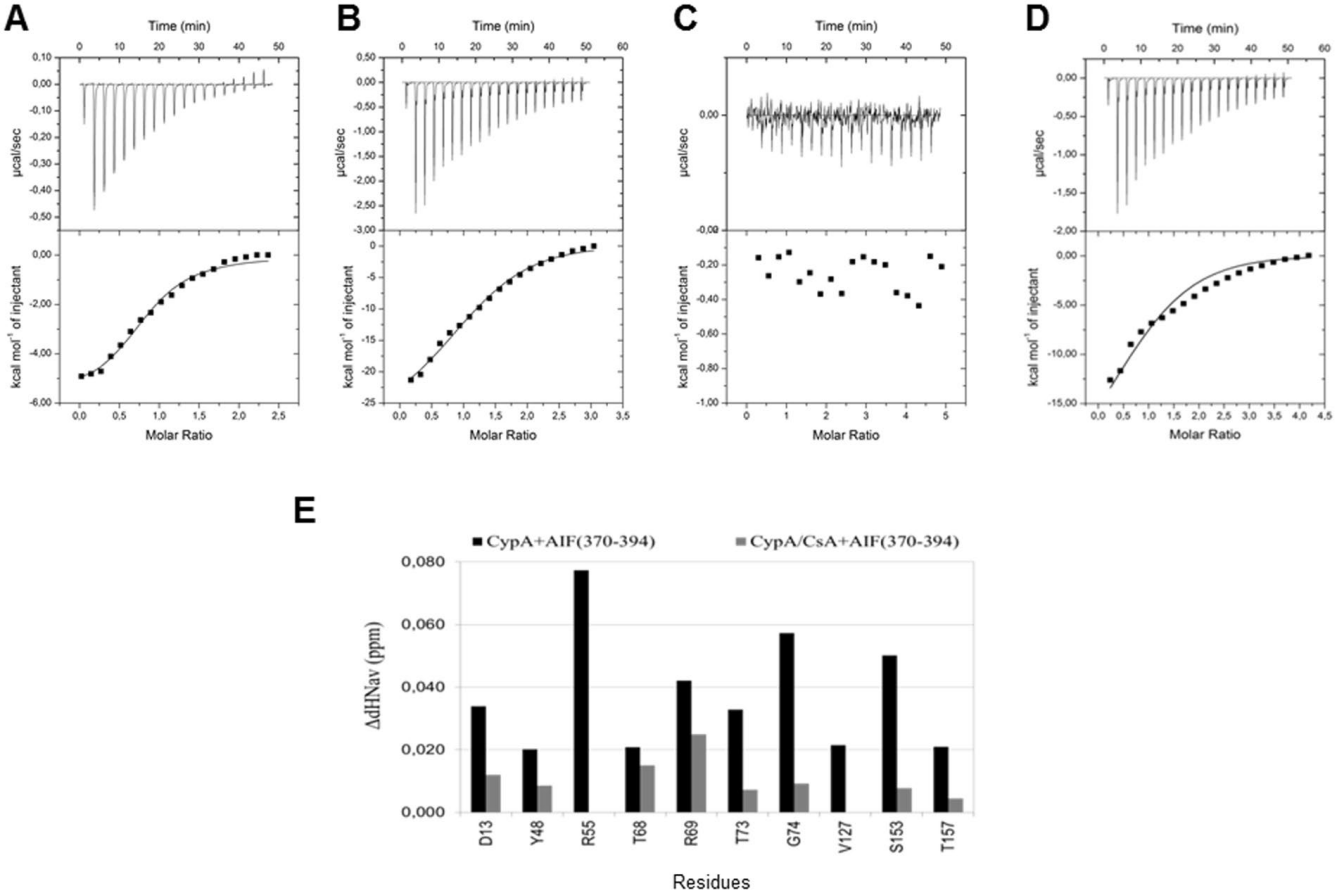
ITC binding assay. (**A**,** B**) Calorimetric titration of AIF(370-394) and CsA with CypA, respectively. The top panels show the heat signal for AIF(370-394) (**A**) and CsA (**B**) injections into a cell containing CypA at 25 °C. The bottom panels show the integrated heat of each injection after correction for the heat of dilution of ligands. Curves represent the best fit to a single-site binding model. (**C**,** D**) ITC competition assays. (**C**) Calorimetric titration of the preformed complex CsA-CypA with AIF(370-394). The top panel shows the heat signal for AIF(370-394) injections into a cell containing CypA/CsA complex at 25 °C. (**D**) Calorimetric titration of the AIF(370-394) - CypA complex with CsA. The top panel shows the heat signal for CsA injections into a cell containing CypA/AIF(370-394) complex at 25 °C. (**E**) Bar graphs of the ΔHN_av_ as a function of the amino acid residue upon addition of AIF(370-394) to the free CypA (black) and the complex CypA/CsA (gray).

This tricky issue was further evaluated by the analysis of the interactions of CypA with both AIF(370-394) and CsA by ITC. Data show that the binding of AIF(370-394) to CypA is exothermic, resulting in negative peaks in the plots of power versus time (Fig. 5A, upper panel). Signals well fit with a single-site binding model and allow the determination of the K_D_, and of enthalpy (ΔH) and entropy (ΔS) changes associated to the interaction (Fig. 5A, lower panel). Data also show that AIF(370-394) binds CypA with a K_D_ of about 6 μM, in good agreement with the value estimated by SPR^18^. Moreover, ITC data show that the interaction is enthalpically driven although there is a slight positive entropic contribution (Table 2). This finding reflects the contributions of Van der Waals, hydrogen bond, and electrostatic interactions to the binding, in agreement with the mainly polar and charged features of the AIF(370-394) binding surface.

**Table 2:** Thermodynamic parameters associated with the binding of CypA with CsA and AIF(370-394).

	Single Site Binding Model				
	N^α^	ΔG^β^	ΔH^β^	TΔS^β^	KD^γ^
CypA-AIF(370-394)	1.0	−7.3	−6.2	1.1	6
CypA-CsA	1.0	−17.8	−29.6	−11.8	0.6
CypA + AIF(370-394)/CsA	1.4	−14.5	−14.7	−0.2	1.8

^α^Number of binding site, ^β^Expressed in kcal/mol, ^γ^Expressed in μM.

By ITC, we estimated a K_D_ value of 0.6 μM for the interaction between CypA and CsA (Fig. 5B). In agreement with previous reports, the binding is underpinned by a stoichiometry of 1:1, is entropically unfavorable and is entirely driven by the exothermic binding enthalpy^26^. By comparing the ITC data related to the two interactions, we notice some interesting features. Beside the remarkable difference in free energies (Δ*G*) that reflects the significant difference in K_D_ values (Table 2), we also note that the binding enthalpy for AIF(370-394) is 10 kcal/mol lower than that of CsA. This difference can be attributed, at least in part, to a greater number of H bond and Van der Waals interactions present in the CypA/CsA complex compared to that formed by CypA and AIF(370-394). Such a difference yields a larger entropic loss in the case of CsA binding (Table 2), indicating that the heat release is accompanied by a strong loss of disorder mostly due to the conformational restriction of the ligand backbone in the cavity. On the contrary, the entropy gain observed in the AIF(370-394)/CypA association might be attributed to a significant electrostatic steering and to a substantially unaltered conformational freedom of the ligand following complex formation, deriving from the reduced affinity and binding interface intervening between AIF(370-394) and CypA.

In competition experiments, when AIF(370-394) was titrated onto the CypA-CsA preformed complex, no apparent heat exchange was observed in a AIF(370-394) concentration range spanning from 5 to 500 μM (Fig. 5C). On the contrary, when CsA was added to the CypA/AIF(370-394) complex, robust heat changes were observed (Fig. 5D). The binding isotherm for CsA, derived from the integrated heat data, could be fitted by a model of a single binding site with an apparent dissociation constant of 1.6 ± 0.5 × 10^−6^ M. Such observations suggest that AIF(370-394) is unable to bind CypA in the presence of CsA, a stronger CypA ligand, while CsA binds CypA displacing AIF(370-394), in agreement with the idea of partially overlapping binding sites. Moreover, the ligand-binding measurements are consistent with a negative cooperation model in which the CsA affinity for CypA is weaker in the presence of AIF(370-394).

To explore in more detail this aspect we performed NMR competition experiments in which AIF(370-394) was gradually added to the preformed CypA/CsA complex. As shown in Fig. 5E, CsA binding to CypA did not fully abrogate the AIF(370-394) interaction with CypA. However, occupancy of the binding site by CsA makes inaccessible V127 and the crucial residue R55. Overall, data indicate that R55 is not crucial for the interaction between CypA and the peptide.

### Docking model of the AIF(Δ1-121)/CypA complex

Data were used to generate an experimentally driven structural model of the AIF(Δ1-121)/CypA. CSP data from NMR titration experiments between CypA and AIF(Δ1-121) proteins were employed to define a set of active and passive residues for HADDOCK calculations^27^. In addition, residues of the region 370-394 of AIF(Δ1-121) exposed to the solvent in the crystal structure, were used to restrict the interaction interface of AIF(Δ1-121).

**Figure 6:**
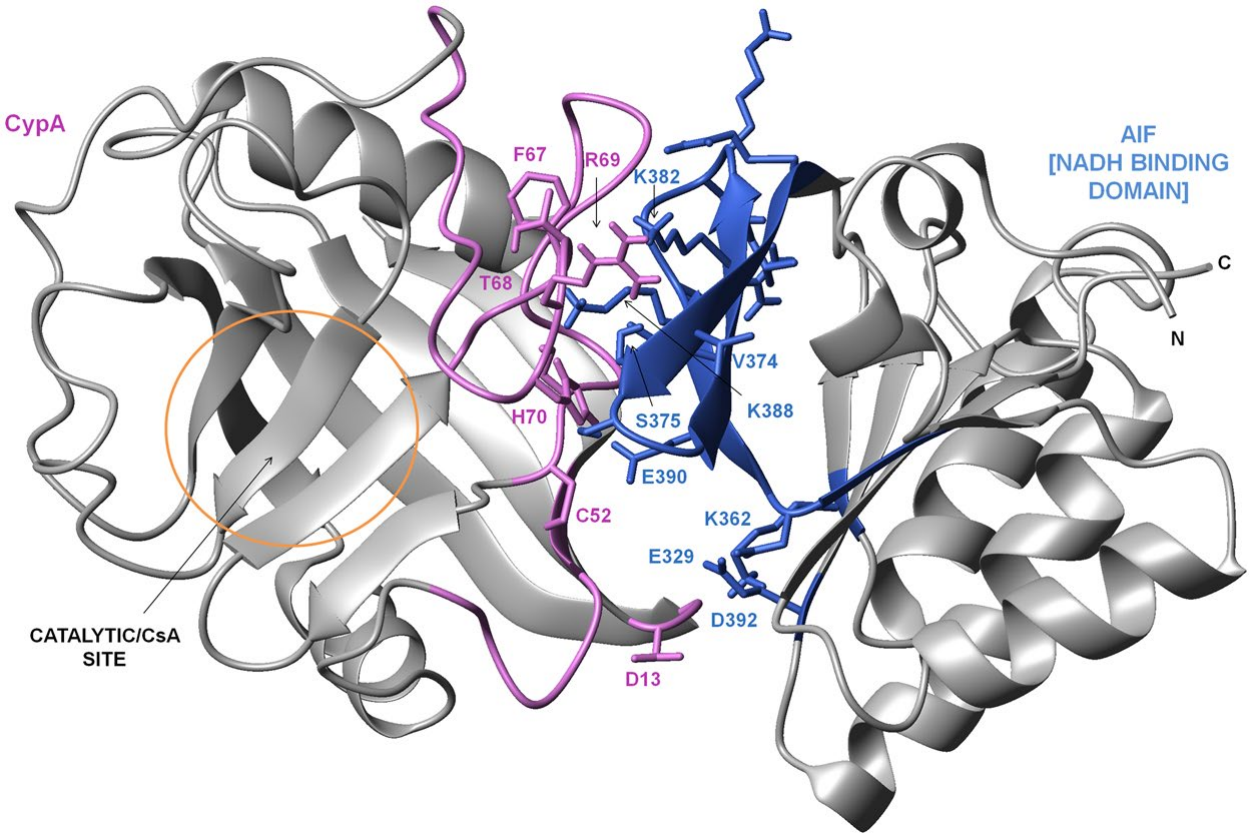
Molecular docking studies. Model of CypA/AIF(Δ1-121) complex provided with the lowest/best Haddock score. The binding regions and side chains of binding residues of CypA and AIF are indicated in violet and royal blue, respectively.

The docking calculations generated 100 solutions that were sorted into 12 clusters based on the HADDOCK score. The best 4 structures of clusters with negative Z-Score were visually inspected. The first cluster with the lowest HADDOCK score was the most populated (46 structures) and resulted more in line with experimental data. The best structure of the first cluster chosen as the representative model of the AIF(Δ1-121)/CypA complex, is shown in Fig. 6.

In this model, which is consistent with structural and biochemical data, main interactions involve loops α1-β3 (G42-C52) and β4-β5 (G65-S99) of CypA. These regions establish contacts with AIF residues 370-392, in agreement with binding studies. In particular, R69 provides a crucial contribution to the binding, establishing hydrogen bond and Van der Waals interactions with V374 and S375 of AIF(Δ1-121), in line with NMR, MS and binding studies using the Ala-mutated CypA-peptides. Furthermore, CypA residues showing exchange contribution, such as C52, T68, H70 (coloured in royal blue in Figs 1 and 2)^28^ were directly involved in the interaction with AIF(Δ1-121).

Our interaction model also explains the difference of affinity displayed by CypA(3-27) for AIF(Δ1-121) and AIF(370-394) (see Table 1). Indeed, additional contacts are predicted between residues of the CypA loop β1-β2 (D13-G14) and of AIF(Δ1-121) not included in the 370-394 region, such as E329 (strand β12), K362 (strand β13), together with D392 (loop β16-β17) (Fig. 6).

Residues of the loop β5-β6, sheet β6 and loop β6-α2 (A101, N102, A103, S110, Q111, C115, T119) are not directly involved in the AIF binding site on CypA in the representative model, though these residues, identified in NMR-CSP experiments, could be indirectly affected by AIF binding. Among the other solutions provided by HADDOCK, there is an interaction model in which CypA contributes to the binding also with these residues, suggesting an additional, lower-affinity, binding site of AIF(Δ1-121) on CypA, close to loops α1-β3 and β4-β5, the primary site recognized by AIF(Δ1-121).

Interestingly, the complex most representative model shows R55 of CypA as not included in the binding interface, suggesting slight differences between AIF(Δ1-121) and AIF(370-394) binding mode on CypA.

Our experiment-based docking model of the AIF/CypA complex delineates a new interaction surface for AIF(Δ1-121) on CypA. This site represents a new specific druggable region of CypA, beside the catalytic pocket. Indeed, a computational analysis of druggable sites on CypA using the FTMap algorithm (see Supporting Information for details)^29^, showed that loops α1-β3 (G42-C52) and a portion of loop β4-β5 (G65-S99), delimit a region with high chances to host ligands with low-micromolar affinity (Supplementary Fig. S9).

## Discussion

The CypA/AIF(Δ1-121) complex is considered a very promising target for compounds with neuroprotective activity^8–18^. Therefore, understanding the molecular mechanism underpinning the CypA/AIF(Δ1-121) recognition may have a broad impact on the development of novel molecular therapies for neurodegeneration.

In this study, we provide the first structural and biophysical characterization of the CypA/AIF(Δ1-121) interaction. We have used both the AIF(Δ1-121) protein and the synthetic peptide encompassing region 370-394, which binds CypA and has been successfully utilized for elucidating the biological implication of CypA in the AIF-mediated neuronal cell death^18^.

The binding surface of AIF(Δ1-121) on CypA, mainly includes loops α1-β3 (G47, S51), β4-β5 (F67, R69, T73, G74, K76, I78, K82, E86, G96) and β5-β6 (A101, N102, S110, Q111). This surface contains, almost entirely, the interaction region of the AIF(370-394) peptide on CypA, in agreement with the peptide’s ability to prevent the CypA/AIF(Δ1-121) binding, and confirming that it might be used as a simplified model to study the interaction.

Interestingly, the identified interaction surface, partially overlap with the catalytic and CsA binding sites on CypA. Moreover, somewhat unexpectedly, for the peptide we found that it interacts with the highly conserved CypA catalytic residue R55, which is also engaged by CsA^24^. However, unlike CsA, AIF(370-394), as well as AIF(Δ1-121), does not impair the catalytic activity of CypA^13, 18^. Additional data obtained with the R55A mutated CypA demonstrate that the interaction of the peptide with the catalytic residue is of limited importance compared to CsA and to CypA substrates, since recognition is substantially retained in the mutated protein and peptide binding is prevented in the presence of strong interacting substrates. This observation clearly indicates that R55 is not required for efficient recognition of the two proteins but incidentally interacts with the AIF(370-394) peptide because of its higher flexibility.

Our studies also demonstrate that the CypA region spanning residues G50-R69, are those most involved in the interaction with AIF, and that residue R69 is crucial for complex formation, delineating the recognition surface of AIF(Δ1-121) on CypA. The identification of these residues provides new insights for the design of new AIF-based peptides or small molecules that have the potential to inhibit the AIF pro-apoptotic action.

The structural information we have gained by mapping the binding surface of AIF(Δ1-121) on CypA by NMR experiments, enables us to propose a new model representative of the AIF(Δ1-121)/CypA complex (Fig. 6). By this model CypA binds AIF(Δ1-121) in a hydrophilic region, very close to the catalytic and CsA binding site including loops α1-β3 (G42-C52) and β4-β5 (G65-S99). The proposed CypA binding interface accommodates very well residues included in the AIF region 370-394, but further extends to E329 on strand β12 and K362 on strand β13, which are spatially close and exposed on the protein surface (Fig. 6)^28^. In this model CypA residue R69 is crucial for the recognition, establishing hydrogen bond and Van der Waals interactions with V374 and S375 of AIF(Δ1-121), in agreement with NMR, MS and binding studies. This should be one of the most important hot spots for structure-based drug design.

The model proposes that the catalytic residue R55 of CypA is not directly involved in the interaction with full length AIF and that the interaction of residues 370-394 of AIF with CypA is slightly different when they are in the isolated peptide or embedded in the protein architecture. This is in agreement with evidences from NMR-CSP and binding experiments. Indeed, no cross-peak for R55 are observed during NMR titration experiments with the proteins, whereas strong perturbations are observed using the synthetic peptide AIF(370-394). We also find that replacement of R55 with alanine in CypA(55-69) completely abrogates the affinity with AIF(370-394), while it only slightly reduces that with AIF(Δ1-121). Data are not surprising as the isolated CypA peptide, because of its higher flexibility in solution, might show an incidentally increased ability to bind the peptide.

Some CypA regions suggested by our data as potential sites of recognition for AIF, do not fit with this model. However, additional contacts have been suggested by the docking computational solutions, like the one involving loop β5-β6 (A101, N102, S110, Q111) of CypA. On the basis of this evidence, we speculated that this region could be a second binding site for AIF, with a weaker affinity compared to that involving α1-β3 and β4-β5. However, NMR data can be explained by an allosteric effect of this region upon the AIF binding. Indeed, this area falls within the “dynamic cluster” recently proposed by Doshi *et al*.^21^, by NMR and molecular dynamic studies on CypA. In particular, they found that the substrate binding in the catalytic cleft provides strong dynamic contacts in regions distal from the site, spanning residues 85-150, and only limited effects in the active site region. Our NMR results then provide further experimental evidence on the binding mode of CypA, probably at the basis of the promiscuity of the enzyme.

In conclusion, our structural and biochemical studies afford the first experimental insights underlying the AIF(Δ1-121) and AIF(370-394) binding to CypA and identifies region 55-69 of CypA as the most involved in the protein-protein interaction. It also provides an explanation of why the peptide and the protein do not inhibit the *cis-trans* peptidyl-prolyl-isomerase activity of CypA. Our data also suggest the occurrence of a second lower affinity site around residues 121-131 and that AIF binding induces a conformation rearrangement on the most flexible residues 95-115.

## Methods

### Peptide synthesis

Peptides were synthesized, purified and characterized as described previously^30^. Peptide concentrations, missing of Trp and Tyr residues, were determined via the Scopes method in which the absorbance of the peptide bond at 205 nm is monitored by using a NanoDrop200c UV-Vis spectrophotometer (Thermo Scientific)^31^.

### Protein preparation

His-tagged CypA (hereafter CypA) and the corresponding R55A mutant, were expressed and purified as previously reported^25^. The protein concentration was determined by reading the absorbance at 280 nm with a NanoDrop200c UV-Vis spectrophotometer and using a theoretical molar extinction coefficient of 8730 M^−1^cm^−1^. ^15^N-labeled protein was prepared by growing bacteria in a minimal medium with ^15^NH_4_Cl (Sigma Aldrich) as the sole nitrogen source, following indications reported in literature^28^. In particular, a single clone of CypA strain was cultured for 16 h at 37 °C in 50 mL of LB medium with antibiotics. The harvested cells were centrifuged for 15 min at 5000 rpm and suspended in 10 mL of M9 medium. Subsequently a spectrophotometric reading of cells was carried out to determine the start value of OD_600nm_, (generally 0.15 ÷ 0.18 absorbance). Cells were then inoculated in M9 medium containing ^15^NH_4_Cl (Sigma Aldrich 1 g/L) as the sole nitrogen source, and grown at 37 °C. For the overexpression, cells were grown at 37 °C till the mid log phase (OD_600_ = 0.9 ± 0.5 absorbance). At this point the expression was induced by addition of IPTG (1 mM). Cells were allowed to grow for further 15-16 hours at 25 °C. After harvesting, cells were lysed and ^15^N-6His-CypA (hereafter ^15^N-CypA) was purified by affinity chromatography on a His-trap HP column, followed by a step of size exclusion chromatography. CypA^R55A^ was prepared starting from the construct pCMV6-AC-Myc-CypA^R55A^ kindly provided by the group of Dr. Valentina Bonetto of the Department of Molecular Biochemistry and Pharmacology-IRCCS “Mario Negri”, Milano, Italy.

### Isothermal Titration Calorimetry (ITC) experiments

ITC experiments were performed at 25 °C using a MicroCal ITC200 (GE Healthcare Bio-Sciences AB, Sweden). Protein and peptide samples were dialyzed in phosphate saline buffer (PBS 1X) containing 1 mM DTT, pH 7.4. In each titration, 20 injections of 2 μL each of AIF(370-394) at 0.7 mM peptide were performed on a sample of 300 μL of CypA at 5 μM. For CypA-CsA experiments, 300 μL of a 20 μM solution of CypA were titrated by adding 2 μL aliquots of a 2 μM CsA solution at 25 °C in PBS buffer containing 0.1% DMSO. Data were analyzed using the “Origin” software (MicroCal). The dissociation constant (KD), molar binding stoichiometry (n) and the binding enthalpy (ΔH), entropy (ΔS) and Gibbs free energy (ΔG) were determined by fitting the binding isotherm to a one-site model with MicroCal Origin software. All ITC experiments were performed in triplicate.

### NMR spectroscopy

NMR experiments were carried out following procedures previously reported^32^. In particular, experiments were developed at 25 °C using an Inova 600 MHz spectrometer (Varian Inc., Palo Alto, CA, USA), equipped with a cryogenic probe optimized for ^1^H detection. NMR samples were concentrated to 400 µM for the assignment of backbone amide in PBS pH 5.8 and ~80 μM for HSQC-based titration experiments in PBS pH 5.8 or 20 mM Tris-d_11_, 100 mM NaCl with 10% D_2_O and 0.02% NaN_3_, pH 7.2. NMR data were processed by the software VNMRJ 1.1.D (Varian Inc.). One-dimensional (1D) spectra were analyzed using ACD/NMR Processor 12.0 [ACD/NMR]; two and three-dimensional (2D and 3D) spectra were analyzed using tools available in CARA (Computer Aided Resonance Assignment) software (downloaded from cara.nmr.ch)^33^.

Almost complete H^N^ and N resonance assignments of ^15^N-HSQC-detected residues of CypA were achieved, as previously reported^34^, based on 3D ^15^N-edited NOESY^35^ and ^15^N-edited TOCSY spectra^36^, acquired with a mixing time of 80 and 50 ms, respectively, in combination with previous assignments reported in literature^37^. The indole H^N^ group of the single Trp residue was unambiguously assigned. To characterize the AIF binding site, intensity reduction of the amide cross-peaks in the CypA ^15^N-HSQC spectrum was evaluated calculating the following ratio: I_bound_/ I_free_^38, 39^ for each residue having well visible cross-peaks in the HSQC spectrum of the free CypA. I_free_ and I_bound_ are, respectively, the amide cross-peak intensities in the absence and in the presence of AIF at 1:0.6 ratio CypA:AIF(Δ1-121). Lower than the mean value minus 1 SD and 0.5 SD were identified as most significative changes of signal intensities and mapped on the three-dimensional structural of CypA according to a color code (Fig. 1). To define the AIF(370-394) binding site, 2D ^1^H-^15^N HSQC spectra were recorded on ^15^N-CypA (80 μM) in the presence of increasing concentrations of AIF(370-394) peptide ranging from 0 to 800 μM in PBS buffer, pH 5.8. The chemical shift perturbation (CSP) was quantified by average combined chemical shifts between the free form and AIF(370-394)-bound CypA protein using the following equation: ΔδHNav = [((ΔδH^N^)^2^ + (ΔδN/5)^2^)/2]^1/2^ where ΔδH and ΔδN are the chemical shift variations of the amide proton and nitrogen resonances, respectively. Strong, medium and weak CSPs were classified as ΔδHN_av_ > mean + 2 SD, mean + 1 SD < ΔδHN_av_ < mean + 2 SD and mean + 0.5 SD < ΔδHN_av_ < mean + 1 SD, respectively.

### NMR-H/D exchange experiments

For NMR H/D exchange experiments, a sample of ~200 μM of ^15^N-CypA in the absence and in the presence of AIF(370-394) (1:10 molar ratio) was lyophilized from PBS at pH 6.3. Lyophilized samples were first dissolved in 100 μL H_2_O (to avoid potential refolding artifacts) and then in 400 μL D_2_O. Samples were quickly transferred to a 5 mm NMR tube and fast ^1^H-^15^N HSQC spectra (time ~10 min) were collected after 7 min from D_2_O addition. A control experiment was run in which a sample of 200 μM of ^15^N-CypA in PBS at pH 6.3 was first lyophilized and then dissolved in 500 μL of H_2_O.

Amide proton protection factors were determined for both the free and bound protein by calculating the ratios of the intensities of the CypA HSQC cross-peaks after H/D exchange and in H_2_O (H^N^-PF = I_H2O_/I_D2O_). Then, the difference of the H^N^-PFs between the bound and free protein (ΔH^N^-PF = H^N^-PF_bound_ - H^N^-PF_free_) was determined^19^. Residues of CypA, whose ΔH^N^-PF are higher than 2, were considered as buried upon AIF(370-394) binding.

### Enzymatic MS-based foot-printing

For MS-based foot-printing analyses, tryptic hydrolyses were performed by adding TPCK-treated trypsin (1 µg/µL) to aliquots (300 pmol) of CypA (15 µM) in the presence of AIF(370-394) (300 µM) at an enzyme/substrate ratio of 1:50 (w/w) in a final volume of 20 µL, incubating mixtures at 37 °C for 2 h. The same amount of CypA without AIF(370-394) and the peptide alone were also digested as control samples. Protein digestions were blocked by adding 80 µL of aqueous 0.1% TFA. Chymotryptic hydrolyses were performed as described above by adding TLCK-treated chymotrypsin at an enzyme/substrate ratio of 1:250 (w/w) and by incubating the mixtures at 37 °C for 2 h. Following enzymatic digestions, samples were centrifuged (15800 g, 15 min). For matrix assisted laser desorption ionization time-of-flight (MALDI-TOF) MS analyses, an internal standard peptide (molecular mass at m/z 620.36) for spectra normalization was added to mixtures (5:1) and 1 μL of diluted samples was mixed with 1 μL of saturated α-cyano-4-hydroxycinnamic acid matrix solution (10 mg/mL in acetonitrile/water (1:1, v/v), containing 0.1% trifluoroacetic acid). Thus, a droplet of the resulting mixture (1 μL) was placed on the MALDI-TOF micro MX (Waters, Manchester, UK) target plate and dried at room temperature. Once the liquid was completely evaporated, samples were loaded into the mass spectrometer and analyzed. The instrument was externally calibrated using a tryptic alcohol dehydrogenase digest (Waters) in reflectron positive ion mode^40^. All spectra were processed and analyzed using the MassLynx 4.1 software. Relative intensity decrease was considered as significant when at least a 1.5 fold change was observed.

### Molecular docking studies

Docking calculations of CypA and AIF(Δ1-121) were performed by the HADDOCK webserver^23^. The structural coordinates of CypA and AIF(Δ1-121) used as the input were obtained from the most representative NMR structure (model 6, PDB ID: 1OCA)^28^ and from the crystal structure (PDB ID: 1M6I)^41^, respectively. CypA active residues were assigned from CypA-AIF(Δ 1-121) CSP NMR studies (D13, G14, R19, G47, S51, F67, R69, T73, G74, K76, I78, K82, E86, G96, M100, A101, N102, S110, Q111, C115, T119, K133, M136, N137, S153). AIF residues of the region 370-394 of AIF(Δ1-121), exposed to the solvent in the crystal structure, were used to define the AIF active residues (Q370, S371, S375, S376, G377, K378, L380, K382, K384, D385, G386, R387, K388, E390, D392, H393). Passive residues were automatically selected for both proteins by the HADDOCK web server^27^. Default HADDOCK parameters were used throughout the docking calculations. During the rigid body energy minimization 1000 structures were generated. Then the 200 lowest energy structures were used for semi-flexible simulated annealing and explicit water-refinement. Clustering of the final 200 structures was performed by the HADDOCK web server. The complex structures were analyzed by MOLMOL^42^.

## Electronic supplementary material

Supplementary Informations
