# Supplementary material for: Structural and biochemical insights of CypA and AIF interaction
Source: Sci Rep. 2017 Apr 25;7:1138. doi: 10.1038/s41598-017-01337-8 (PMC5430804; doi:10.1038/s41598-017-01337-8)
Supplement: Supplementary file 1 — Supplementary Informations [file 41598_2017_1337_MOESM1_ESM.pdf]

Supplementary Information

Structural and biochemical insights of CypA and AIF interaction

Biancamaria Farina[1], Gianluigi Di Sorbo[1,2], Angela Chambery[2], Andrea Caporale[1], Guido Leoni[3], Rosita Russo[2], Fabiola Mascanzoni[1], Domenico Raimondo[4], Roberto Fattorusso[2], Menotti Ruvo[1] & Nunzianna Doti[1*]

[1]Istituto di Biostrutture e Bioimmagini, C.N.R. and CIRPEB; Via Mezzocannone 16, 80134, Napoli, (Italy). [2]Dipartimento di Scienze e Tecnologie Ambientali, Biologiche e Farmaceutiche, Seconda Università degli Studi Napoli, via Vivaldi 46, 81100, Caserta (Italy). [3]Nouscom s.r.l. via di Castel Romano 100, 00128, Roma (Italy). [4]Sapienza, Università di Roma - Viale Regina Elena 324, 00161, Roma (Italy). Correspondence and requests for materials should be addressed to N.D. (email: nunzianna.doti@cnr.it)

Experimental Section

Direct binding assays

Direct binding assays were performed with the Corning Epic label-free technology with the EnSpire Multimode Plate Reader (PerkinElmer, Rodgau, Germany) as reported in literature.[1] Briefly, AIF(370-394) peptide immobilization on the optical biosensors was accomplished by adding 100 µg/mL streptavidin solution in 20 mM sodium acetate at pH 4.0, for 16 h at 4 °C. The microplate was subsequently washed three times with phosphate-buffered saline (PBS 1X, pH 7.4). After washing, the plate was incubated with a solution of 50 µg/mL of biotinylated AIF(370-394) in the assay buffer (PBS 1X, pH 7.4) for 2 hours followed by three washing steps in PBS 1X at pH 7.4. After 2 h incubation, a baseline reading was recorded. Then 30 µL of peptide samples were dispensed in the plate. The final readings were taken over a period of 1 hour. Label-free responses were measured as shifts in reflected wavelength and were expressed in picometers (Fig. S4). The difference between the last baseline measurements and the maximum signal was used to determine the KD values (Table 1 in the main text). Direct binding assays against AIF(Δ1-121) protein were performed as previously described, but in this case AIF(Δ1-121) was immobilized onto the optical biosensor by adding directly 100 µg/mL of protein in 20 mM sodium acetate pH 4.0, for 16 h at 4 °C (Fig. S5). The immobilization of CypA and $CypA^{R55A}$ onto the optical biosensor was carried out by adding 200 µg/mL of protein in 20 mM sodium acetate pH 5.5, for 16 h at 4 °C. AIF(Δ1-121) protein and AIF(370-394) peptide were added at several concentrations (Fig. S7A and B), following the procedure described above. Results were analyzed using the EnSpire label-free user interface software. Plots were generated using GraphPad PrismR V-5.01 (GraphPad Software, La Jolla, CA).

FTMap algorithm

The FTMap algorithm was used in order to predict CypA binding “hot spots”, i.e., regions of the protein surface with major contributions to ligand-binding free energy. The strategy of FTMap uses individual functional groups or small organic molecules to probe the protein surface identifying regions that are capable of binding multiple ligands. At a few locations of the protein, multiple highly ranked probe clusters can be seen to overlap, forming consensus clusters (CCs) that identify the binding hot spots. It has been demonstrated that FTMap approach is effective to provide robust prediction of binding hot spots, with excellent agreement with experimental data and that the fragment hit rate predicts the importance of the site. In particular, the sites on the protein known to be druggable invariably contain a strong hot spot with 16 or more probe clusters (PMID: 19176554,

17305325, 25855957) [2-4]. In our study, the mapping of the apo structure of CypA (PDB ID 1bck; PMID: 9769216)[5] yields the CCs CS000 (23 probe clusters), CS001 (19 probe clusters), C002 (16 probe clusters) and CS003 (11 probe clusters), CS004 (7 probe clusters), CS005 (4 probe clusters), CS006 (4 probe clusters) and CS007 (4 probe clusters), CS008 (3 probe clusters), CS009 (2 probe clusters). The top scoring ligand-binding site identified by FTMap on CypA is the active site, while the second, CS001 (Figure S7) is constituted by loops connecting α1-β3 (G42-C52) and a portion of the loop connecting β4-β5 (G65-S99), close to the catalytic site of CypA within the same interaction surface identified by NMR studies and mapped by trypsin MS-based foot-printing and contains 19 probe clusters. This result shows that a second druggable region (beyond the active site) based on ligand-free protein structures, can be found on the CypA protein surface very closed to the catalytic site.

Fluorescence-based assays

Fluorescence quenching assays were performed using the FRET substrate Ac-EK(Dabcyl)PPFAE(EDANS)KA-NH2 (MW 1556.2 amu) reported in literature.[6] At 0.23 ng/µL (0.15 µM) the substrate provides a significant fluorescence emission at 510 nm compared to the buffers used in the assay (PBS 1X) (Figure S8A). Then, the substrate at this fixed concentration was titrated in 100 µL wells of 384-micro plates with different amount of CypA and $CypA^{R55A}$ (in the range between 7.2 nM to 1.75 µM). Kinetic constants were determined by fitting data of fluorescence maxima at 510 nm of EDANS upon protein additions. Results are presented as percent fraction of quenched substrate and represent the mean ± SD versus CypA (continuous line) and $CypA^{R55A}$ (dotted line) concentrations (Figure S8B). Competitive effects of AIF(370-394) and CsA (Fig. S8B, red and blue lines, respectively) on the CypA/substrate complex formation were determined using a 10-fold excess (1.5 µM) of compounds.

References

1 Oppermann, S., Schrader, F. C., Elsässer, K., Dolga, A. M., Kraus, A. L., Doti, N., Wegscheid-Gerlach, C., Schlitzer, M. & Culmsee, C. *J Pharmacol Exp Ther* **350,** 273-289 (2014).

2 Kozakov, D., Grove, L. E., Hall, D. R., Bohnuud, T., Mottarella, S. E., Luo, L., Xia B., Beglov D. & Vajda, S. *Nat Protoc* **10,** 733-755 (2015).

3 Brenke, R., Kozakov, D., Chuang, G. Y., Beglov, D., Hall, D., Landon, M. R., Mattos, C. & Vajda, S. *Bioinformatics* **25,** 621-627 (2009).

4 Landon, M. R., Lancia D. R. Jr., Yu, J., Thiel, S. C. & Vajda S., *J Med Chem* **50,** 1231-1240 (2007).

5 Kallen, J., Mikol, V., Taylor, P. & Walkinshaw, M. D. *J Mol Biol* **283,** 435-449 (1998).

6 Caporale, A., Mascanzoni, F., Farina, B., Sturlese, M., Di Sorbo, G., Fattorusso, R., Ruvo, M. & Doti, N. *J Biomol Screen* pii: 1087057116650402 (2016).

Figure S1

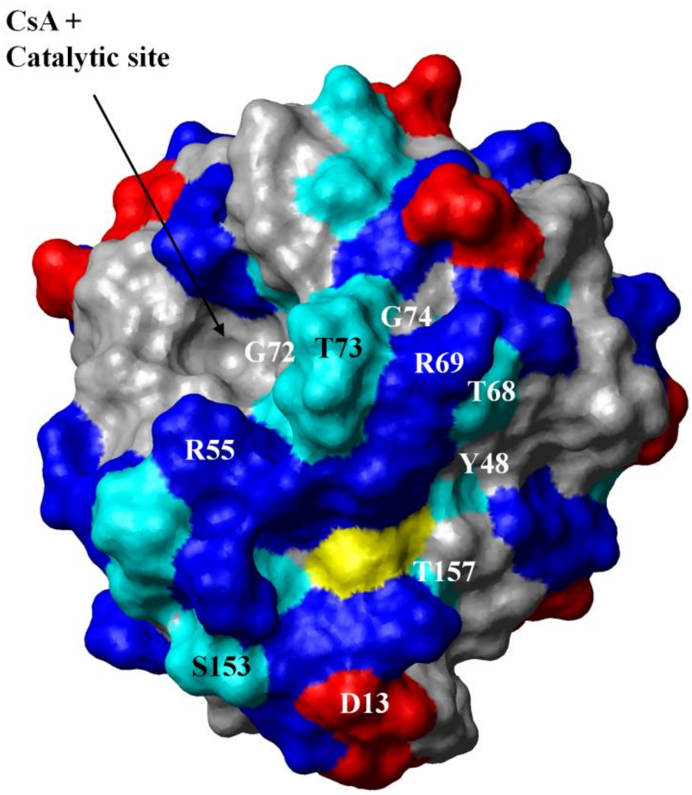

Figure S1. Distribution of positively (blue) and negatively (red) charged, polar (cyan) and hydrophobic (gray) residues on the CypA surface encompassing the AIF(370-394) binding site. Residues affected by AIF(370-394) interaction observed in the CSP-NMR studies are indicated. Cysteine residues were colored in yellow.

Figure S2

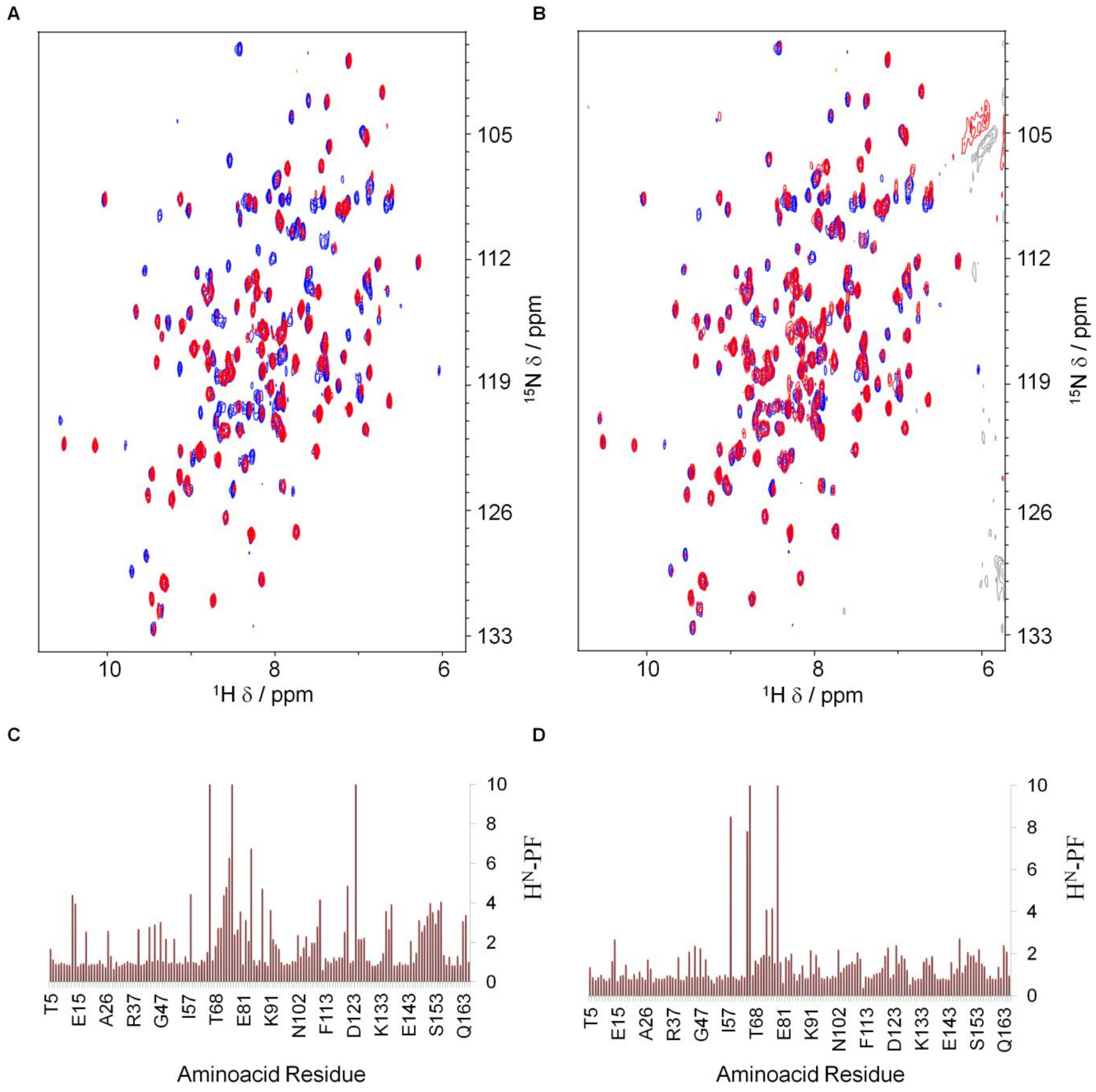

Figure S2. NMR-H/D exchange studies of ^{15}N-CypA in the absence (**A** and **C**) and in the presence (**B** and **D**) of AIF(370-394). Superposition of 2D [^{1}H, ^{15}N] HSQC sections of the free (**A**) and bound CypA (**B**) before (blue) and after D_2O exchange (red). Bar graphs of the amide hydrogen protection factor (H^N-PF) for the free (**C**) and bound CypA (**D**) as function of the amino acid residue.

Figure S3

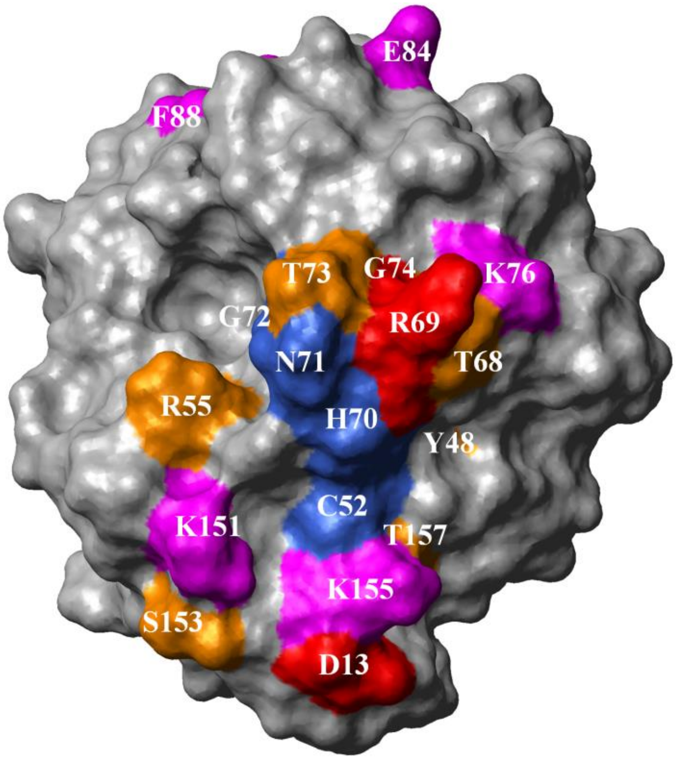

Figure S3. Distribution of CypA residues identified in CSP and H/D-exchange NMR titration experiments using the peptide AIF(370-394) as ligand. Amino acids of CypA that are identified by NMR-CSP studies are shaded in gold yellow, by H/D-exchange in pink and those identified by either experiments in red. Residues scarcely visible in the HSQC (C52, H70 and N71) are painted in navy on the CypA structure.

Figure S4

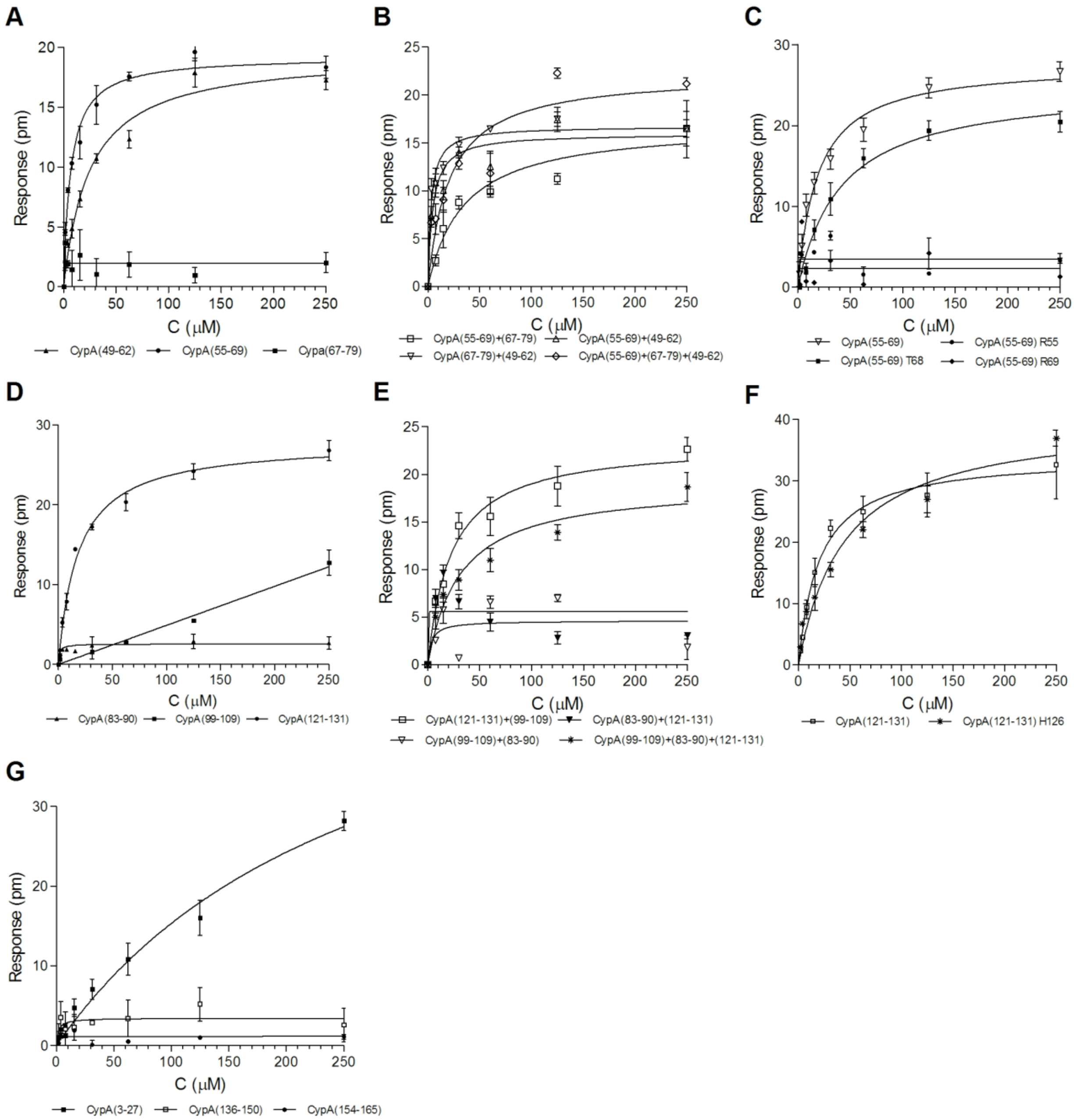

Figure S4. Direct binding assays of CypA-derived peptides, alone (A, D and G), in combination (B and E) and mutated (C and F) with AIF(370-394). All CypA-derived peptides were tested at several concentrations over a surface coated with streptavidin-biotinylated-AIF(370-394) peptide. Results were analyzed using the EnSpire label-free user interface software. Graphs were generated using GraphPad PrismR V-5.01 (GraphPad Software, La Jolla, CA). Results are means of triplicates.

Figure S5

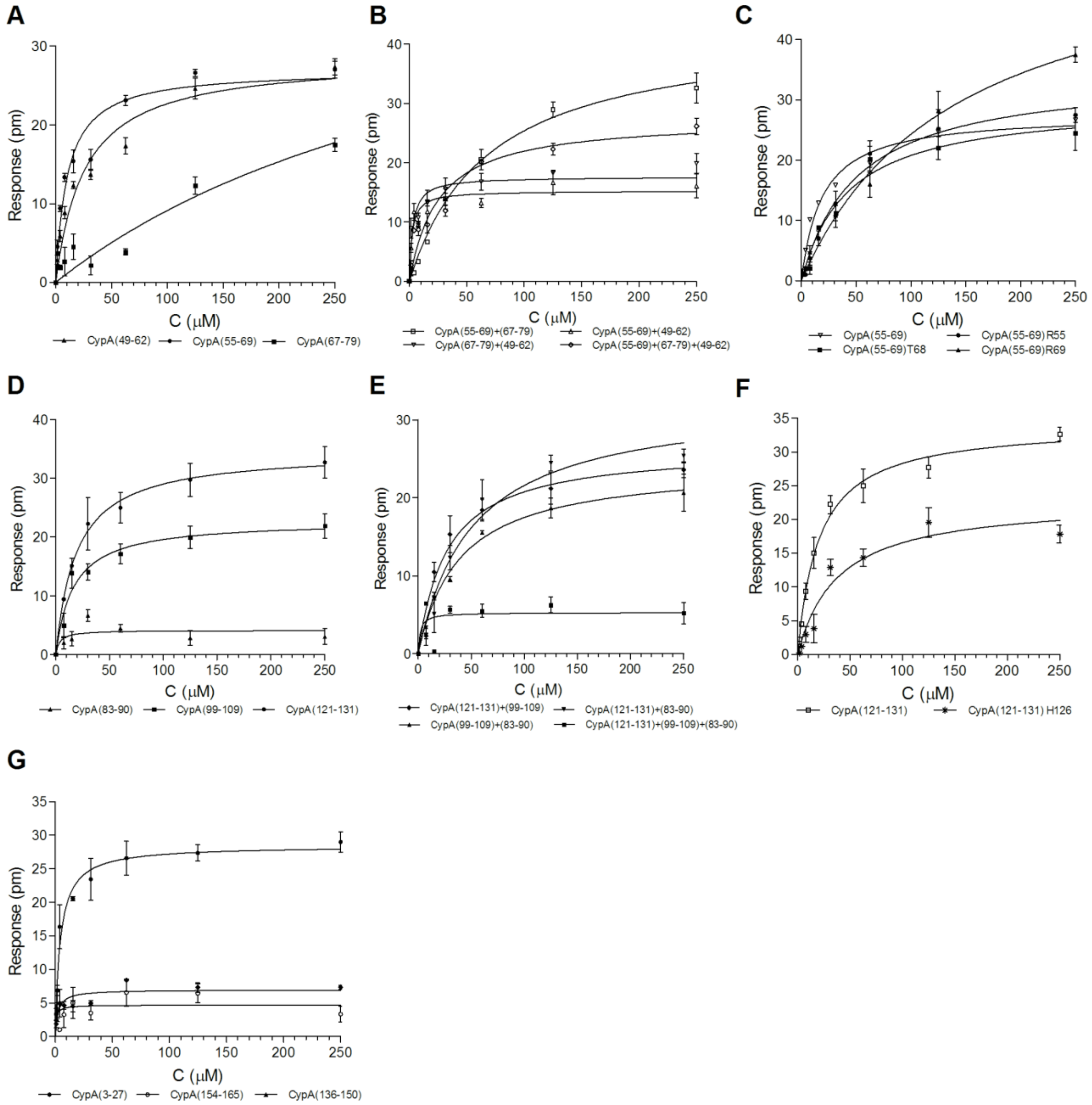

Figure S5. Direct binding assays of CypA-derived peptides, alone (**A**, **D** and **G**), in combination (**B** and **E**) and mutated (**C** and **F**) with AIF(Δ1-121) protein. All CypA-derived peptides were tested at several concentrations over a surface coated with AIF(Δ1-121). Results were analyzed using the EnSpire label-free user interface software. Graphs were generated using GraphPad PrismR V-5.01 (GraphPad Software, La Jolla, CA). Results are means of triplicates.

Figure S6

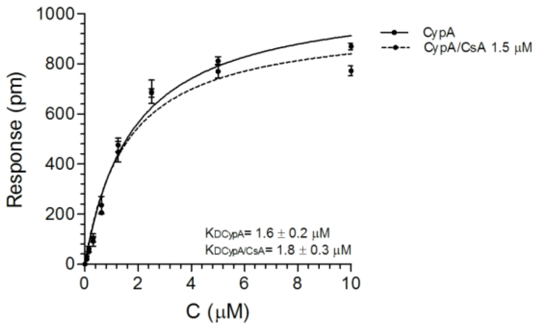

Figure S6. Direct binding assays. AIF(Δ1-121) was tested at several concentrations over a surface coated with CypA wild type with and without the CsA. Results were analyzed using the EnSpire label-free user interface software. Graphs were generated using GraphPad PrismR V-5.01 (GraphPad Software, La Jolla, CA). Results are means of triplicates.

Figure S7

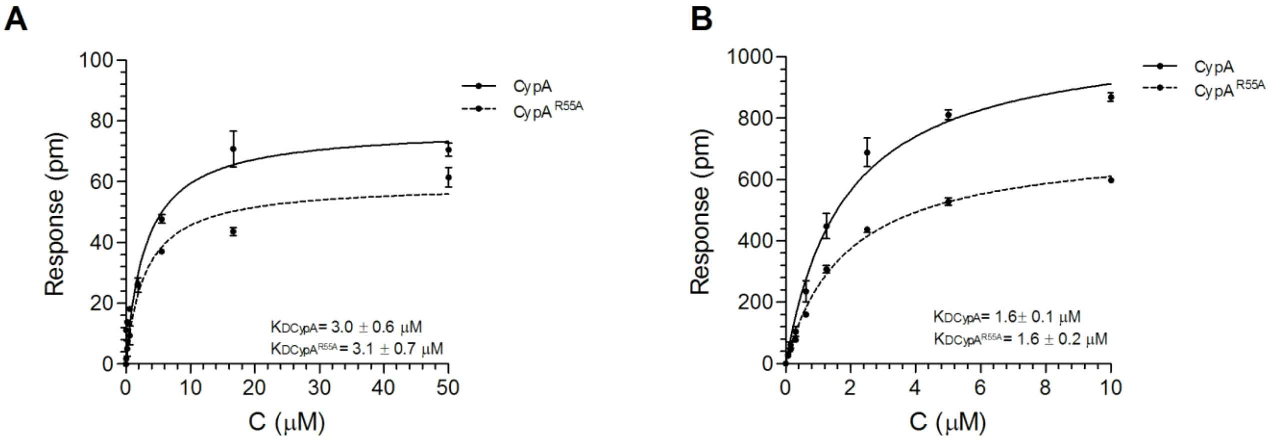

Figure S7. Direct binding assays of CypA and $CypA^{R55A}$ vs AIF(370-394) (**A**) and AIF(Δ1-121) (**B**). Results were analyzed using the EnSpire label-free user interface software. Graphs were generated using GraphPad PrismR V-5.01 (GraphPad Software, La Jolla, CA). Results are means of triplicates.

Figure S8

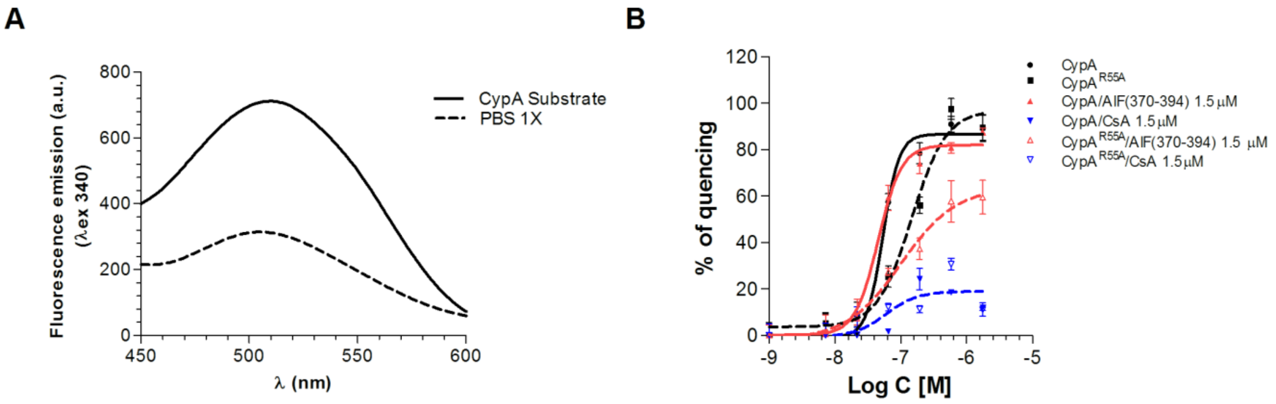

C

Best-fit values of EC50 (M)			
	**Sub**	**Sub/AIF(370-394)**	**Sub/CsA**
**CypA**	5.4 ± 0.7 *10^{-8}	4.6 ± 0.6 *10^{-8}	Not converged
**CypA^{R55A}**	1.5 ± 0.8 *10^{-7}	1.1 ± 0.9 *10^{-7}	Not converged

Figure S8. Binding assay based on fluorescence quenching. (**A**) Fluorescence emission spectrum of CypA substrate. Samples were excited at 340 nm, and the emission in the range of 450-600 nm was measured. A significant difference (Δfluorescence emission = 319 a. u.) in the fluorescence emission at 510 nm was detected between the substrate (continuous line) and the buffer (dotted line). (**B**) Dose-response curves of fluorescence quenching of substrate with increasing concentration of CypA, CypA^{R55A} and of both proteins incubated with the peptide AIF(370-394) and CsA at 1.5 μM. (**C**) EC50 values were determined by using the sigmoidal dose-response fitting of GraphPad PrismR V-5.01. Results are means of triplicates.

Figure S9

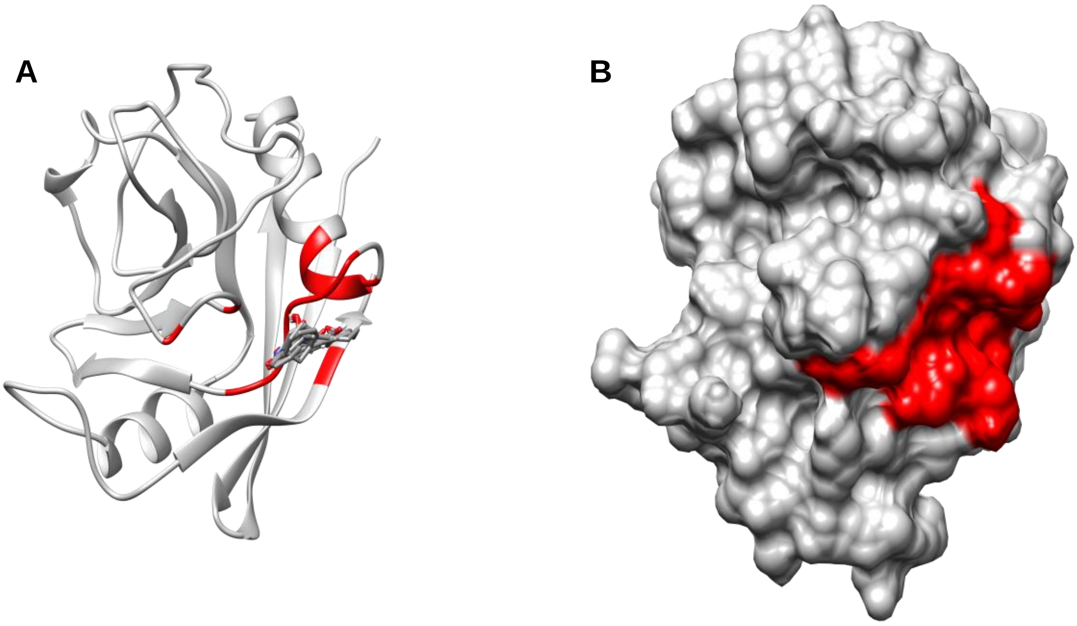

Figure S9. (**A**) Druggable region (beyond the active site) identified by FTMap on CypA and defined by loops connecting α1-β3 (G42-C52) and a portion of the loop connecting β4-β5 (G65-S99). The region is reported as red ribbon in (**A**) while the site has been depicted as red surface in (**B**).
